# Randomized Controlled Trial of Physical Exercise in Diabetic Veterans With Length-Dependent Distal Symmetric Polyneuropathy

**DOI:** 10.3389/fnins.2019.00051

**Published:** 2019-02-11

**Authors:** Evan B. Stubbs, Morris A. Fisher, Clara M. Miller, Christine Jelinek, Jolene Butler, Conor McBurney, Eileen G. Collins

**Affiliations:** ^1^Research Service, Department of Veterans Affairs, Edward Hines Jr. VA Hospital, Hines, IL, United States; ^2^Department of Ophthalmology, Stritch School of Medicine, Loyola University Chicago Health Sciences Division, Maywood, IL, United States; ^3^Department of Neurology, Stritch School of Medicine, Loyola University Chicago Health Sciences Division, Maywood, IL, United States; ^4^Neurology Service, Department of Veterans Affairs, Edward Hines Jr. VA Hospital, Hines, IL, United States; ^5^Department of Biobehavioral Health Science, College of Nursing, University of Illinois at Chicago, Chicago, IL, United States

**Keywords:** diabetes, human, exercise, peripheral nerve, nerve conduction studies, metabolic

## Abstract

**Rationale:** Physical exercise is an essential adjunct to the management of patients with type 2 diabetes mellitus. Therapeutic interventions that improve blood flow to peripheral nerves, such as exercise, may slow the progression of neuropathy in the diabetic patient.

**Aims:** This randomized clinical trial was conducted to determine whether a structured program of aerobic, isokinetic strength, or the combination of aerobic–isokinetic strength exercise intervention alters peripheral nerve function in glycemic-controlled diabetic patients with advanced length-dependent distal symmetric polyneuropathy.

**Methods:** Forty-five patients with type 2 diabetes mellitus exhibiting tight glycemic control (HbA_1c_ intergroup range 7.2–8.0%) were randomized by block design across four experimental groups: sedentary controls (*n* = 12), aerobic exercise (*n* = 11), isokinetic strength (*n* = 11), or the combination of aerobic–isokinetic strength training (*n* = 11). Patients randomized to training groups exercised 3× per week for 12 weeks, whereas patients randomized to the sedentary control group received standard of care. To minimize attention and educational bias, all patients attended a 12-session health promotion educational series. At baseline, immediately following intervention, and again at 12-week post-intervention, detailed nerve conduction studies were conducted as a primary outcome measure. At these same intervals, all patients completed as secondary measures quantitative sensory testing, symptom-limited treadmill stress tests, and a Short-Form 36-Veterans Questionnaire (SF-36V).

**Results:** Of the 45 patients randomized into this study, 37 (82%) had absent sural nerve responses, 19 (42%) had absent median sensory nerve responses, and 17 (38%) had absent ulnar sensory nerve responses. By comparison, responses from tibial nerves were absent in only three (7%) subjects while responses from peroneal nerves were absent in five (11%) subjects. Eleven (92%) of 12 patients that had volunteered to be biopsied exhibited abnormal levels of epidermal nerve fiber densities. Exercise, regardless of type, did not alter sensory or motor nerve electrodiagnostic findings among those patients exhibiting measurable responses (*ANOVA*). There was, however, a modest (*p* = 0.01) beneficial effect of exercise on sensory nerve function (*Fisher’s Exact* Test). Importantly, the beneficial effect of exercise on sensory nerve function was enhanced (*p* = 0.03) during the post-intervention interval. In addition, three of six patients that had undergone exercise intervention exhibited a marked 1.9 ± 0.3-fold improvement in epidermal nerve fiber density. By comparison, none of three sedentary patients whom agreed to be biopsied a second time showed improvement in epidermal nerve fiber density. Compared to baseline values within groups, and compared with sedentary values across groups, neither aerobic, isokinetic strength, or the combination of aerobic–isokinetic strength exercise intervention altered peak oxygen uptake. Patients that underwent aerobic or the combined aerobic–isokinetic strength exercise intervention, however, demonstrated an increase in treadmill test duration that was sustained over the 12-week post-intervention period.

**Conclusion:** A 12-week course of physical exercise, regardless of type, does not alter sensory or motor nerve electrodiagnostic findings. In a subset of patients, a short-term structured program of aerobic exercise may selectively improve sensory nerve fiber function. Large-scale exercise lifestyle intervention trials are warranted to further evaluate the impact of aerobic exercise on sensory nerve fiber function in diabetic neuropathic patients.

**Clinical Trial Registration:** www.ClinicalTrials.gov, identifier NCT00955201.

## Introduction

According to the Centers for Disease Control and Prevention *National Diabetes Statistics Report 2017*, 23.0 million (7.1%) Americans ages 18 years or older have diabetes mellitus (predominantly type 2) (National Diabetes Statistics Report, 2017). An additional 7.2 million (2.2%) Americans were found to be unaware of, or did not report, having diabetes. Globally, diabetes is reported to affect over 400 million people, or 5.3% of the world population (World population data sheet-Population Reference Bureau, 2017), with an additional estimated 318 million individuals (4.2%) exhibiting impaired glucose tolerance (or pre-diabetes), a known risk factor for developing diabetes mellitus (Goncalves et al., 2018). By the year 2040, an estimated 640 million people will be affected worldwide with this disorder.

Peripheral or autonomic nerve dysfunction is a complex, and sometimes life-threatening, heterogenous complication of chronic diabetes (Pop-Busui et al., 2017). Approximately half of all persons with diabetes develop symptomatic neurologic complications during their lifetime (Young et al., 1993; Javed et al., 2015). Alarmingly, nearly half of all neuropathies that develop in diabetic persons are asymptomatic, increasing risk for injury to affected limbs (Pop-Busui et al., 2017). Persons with pre-diabetes are also at risk for developing neuropathies (Ziegler et al., 2008; Smith and Singleton, 2012; Bongaerts et al., 2013). Typical and atypical diabetic neuropathies (Tesfaye et al., 2010) are associated with considerable morbidity (including debilitating physical and psychosocial comorbidities) and mortality. Nearly a third of patients with diabetes report pain. In as much as 20% of diabetic patients, unremitting painful neuropathy negatively impacts health-related quality of life (Abbott et al., 2011). Collectively, diabetic neuropathy remains a significant adverse challenge for both affected patients and concerned health care providers (Vinik et al., 2013; Alleman et al., 2015). An improved understanding of its manifestations, prevention strategies, and the development of novel treatment interventions is paramount to advancing the clinical management of the diabetic neuropathic patient.

Through improved metabolic and glycemic control, contemporary pharmacological strategies have markedly advanced the clinical care of the diabetic patient (Cefalu, 2012). By reducing development of hyperglycemia-associated microvascular pathologies (Stratton et al., 2000; Fowler, 2008; Boussageon et al., 2011; Martin et al., 2014), these strategies aim to slow the progression of retinopathic, nephropathic, and neuropathic diabetic target-organ complications (Cade, 2008; Chawla et al., 2016). In contrast to diabetic retinopathy and nephropathy, where early diagnostic testing is often systematically applied, the management of associated nerve dysfunction, including refractive neuropathic pain, remains a dynamic challenge for both affected patient and provider (Javed et al., 2015). Whereas many classes of medications (e.g., tricyclic antidepressants, anticonvulsants, antioxidants, serotonin-norepinephrine selective reuptake inhibitors, GABA analogs, opiates, topical TRPV1 agonists) are available to achieve symptomatic relief, only three therapies are FDA-approved in the United States for the management of painful diabetic neuropathy and they do little to prevent or alter the course of the disease. There are currently no licensed pharmacologic therapies available in the United States or United Kingdom for the treatment of diabetic neuropathy. An additional concern involves diabetic autonomic neuropathy. This complication often remains a potentially fatal underdiagnosed clinical complication (Vinik and Erbas, 2013). Innovative treatment strategies designed to slow the progression of pre-existing nerve injury in the aged diabetic patient are critically needed.

Advancements in clinical practice for the early detection of neuropathy, coupled with lifestyle intervention for improved glycemic control, are being intensively investigated as viable non-invasive alternatives to pharmacotherapy (Fisher et al., 2007; Singleton et al., 2014, 2015; Houston et al., 2017; Look, 2017). One such lifestyle intervention that may prove beneficial to preserving/restoration of peripheral nerve function involves physical exercise. It is well established that structured aerobic exercise improves glycemic control in type 2 diabetic patients, largely through a cumulative increase in whole-body insulin sensitivity (van Dijk and van Loon, 2015). Resistance exercise has similarly been associated with improvements in insulin sensitivity and glucose tolerance (Bacchi et al., 2012). Less well understood, however, is the impact that physical exercise elicits on the progression of diabetic complications (Leehey et al., 2016), including neuropathy (Fisher et al., 2007; Knauf and Koltyn, 2014). Experimental studies suggest that aerobic exercise may delay the onset of neuropathic pain in a diabetic animal model (Shankarappa et al., 2011), possibly by improving microvascular dysfunction (Boa et al., 2014; Leardini-Tristão et al., 2017). Alternatively, diabetic patients with painful neuropathy are reported to exhibit a heightened level of pain perception during isometric exercise (Knauf and Koltyn, 2014). In healthy individuals, exercise is associated with attenuation of pain when a painful stimulus is administered during or immediately following an exercise session (Koltyn, 2000).

The primary objective of this study was to determine whether a 12-week (36-session) structured training program of aerobic-, isokinetic strength-, or combined aerobic–isokinetic strength exercise, compared with sedentary non-exercise, would alter peripheral sensory or motor nerve electrophysiologic properties in Veterans with chronic (>10 years) type 2 diabetes and length-dependent distal symmetric polyneuropathy (Fisher et al., 2007). Secondary objectives were to establish whether physical exercise alters responses to quantitative sensory testing, self-reported health status using the standardized SF-36V, or alters peak metabolic or hemodynamic parameters in this sample patient population.

This is the first randomized, controlled trial assessing the effect of different structured exercise programs on peripheral nerve function in diabetic neuropathic patients.

## Materials and Methods

### Patient Selection

**FIGURE 1:**
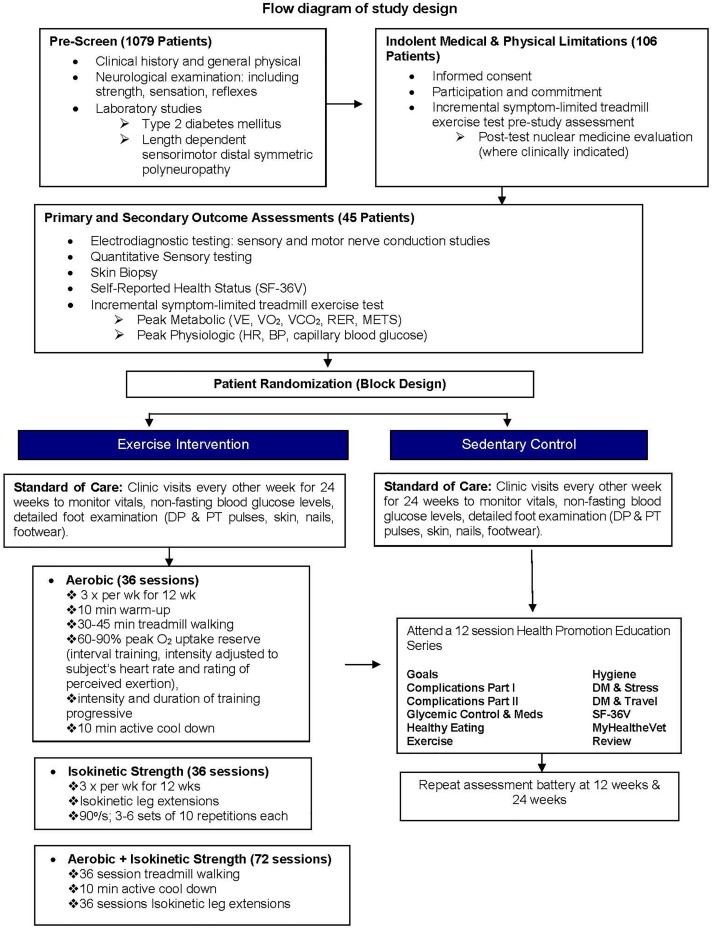
Flow diagram of study design.

From 2010 to 2014, patients aged 45–80 seen at Edward Hines Jr. VA Hospital and surrounding care settings were pre-screened by chart review or in person for enrollment into ClinicalTrials.gov NCT00955201 (Figure 1). Written informed consent was obtained for one hundred six eligible patients. All consented, but not yet randomized, patients completed baseline testing that included a detailed clinical history, physical (including blood pressure monitoring and foot exams), neurological examination with nerve conduction studies, laboratory studies (including fasting blood glucose, HbA_1c_, lipid profile, immune tests), and two incremental symptom-limited treadmill exercise sessions.

**FIGURE 2:**
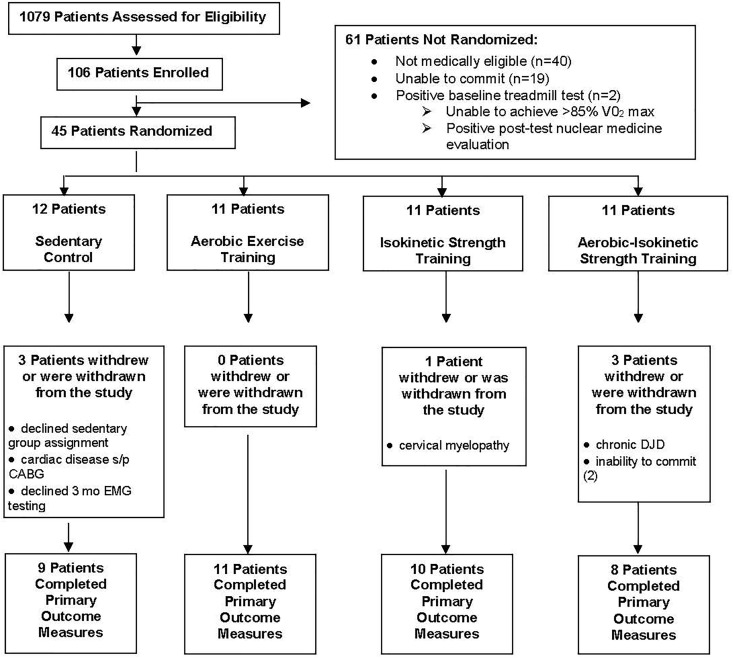
Flow diagram of patient enrollment, randomization, and participation.

Forty-three male and two female patients (*n* = 45) were randomized using *Research Randomizer* by permuted block design (six sets of eight participants per set) across four experimental groups: sedentary controls (*n* = 12), aerobic (*n* = 11), isokinetic strength (*n* = 11), or the combination aerobic–isokinetic strength training (*n* = 11). Subject randomization was blinded to staff (ES and MF) responsible for obtaining and analyzing data on primary and secondary study outcomes. Because of the nature of the study, patients and clinical staff not involved in data analyses were not blinded to the study group assignments. Of the 45 randomized patients, 38 (84%) completed all three timed components (baseline, intervention, 12-week post-intervention) of the primary outcome measure whereas seven patients withdrew or were withdrawn post randomization (Figure 2). Throughout this study, patient compliance with completing secondary outcome measures was variable. No patients experienced a study-related serious adverse event. Nineteen (42%) randomized patients experienced a total of 33 unanticipated serious adverse events unrelated to the study with 15 patients requiring hospitalization.

### Inclusion Criteria

Randomized patients exhibited a fasting plasma glucose concentration ≥126 mg/dL or a 2-h plasma glucose concentration ≥200 mg/dL after a 75 g oral glucose tolerance test, consistent with the American Diabetes Association *Standards of Medical Care in Diabetes-2014* (American Diabetes Association, 2014) and the World Health Organization criteria for diabetes. To normalize against any effect of exercise on glycemic control (van Dijk and van Loon, 2015), only patients with stable levels of glycosylated hemoglobin (HbA_1c_), defined as having ≤1.5% change in HbA_1c_ levels during the previous 6 months, were eligible for inclusion. Patients also exhibited clinical findings consistent with length-dependent sensorimotor distal symmetric polyneuropathy (stage N2a) as defined by positive or negative distal sensory symptoms and nerve conduction abnormalities in at least two distal nerves. Positive sensory symptoms included aching, burning, and tingling while negative symptoms included loss of feeling with diminished ability to distinguish heat from cold in the feet.

### Exclusion Criteria

Screened patients were excluded if they presented with foot ulceration, unstable heart disease, or co-morbid conditions limiting exercise. Patients were also excluded if they had disorders of the central nervous system causing weakness or sensory loss determined by clinical history and neurological examination, had received treatment with medications known to have neuropathy as a prominent side effect including vincristine, vinblastine, *cis*-platin, and paclitaxel, or had medical conditions that may be associated with neuropathies such as alcoholism (ongoing heavy alcohol use by history), liver disease (abnormal liver function tests), kidney disease (elevated creatinine), toxic exposure (by history), vitamin deficiency (by history and laboratory studies as well as clinical signs and symptoms), autoimmune disorders (immunoglobulin abnormalities, studies for collagen diseases), cancer (by history and review of medical record, laboratory studies), or hypothyroidism (increased thyroid stimulating hormone, TSH).

### Incremental Symptom-Limited Treadmill Stress Test

A modified Bruce protocol developed for individuals with peripheral vascular disease and osteoarthritis was used (Langbein et al., 2002). An initial ramp characteristic (5% grade, 2 mph × 6 min) was followed by small increases in percent grade (2.5%) and speed (0.3 mph) thereafter every 2 min until the patient terminated the test. Metabolic measurements (MedGraphics CPX/D system, Saint Paul, MN, United States) were captured breath-by-breath beginning 2 min before and ending 5 min after exercise. Cardiac function was monitored by 12-lead electrocardiographic recording prior to, during, and immediately following each incremental symptom-limited treadmill stress test as detailed below.

### Aerobic or Isokinetic Strength Training

Patients randomized to aerobic-, isokinetic strength-, or the combination of aerobic–isokinetic strength exercise were trained three times weekly. A 10-min warm-up consisting of stretching and flexibility exercises preceded all training sessions. Patients randomized to the combined exercise group received aerobic training prior to isokinetic strength training. A 10-min between session break allowed for the patients’ heart rate to return to within 10 bpm from resting. Exercised training duration and intensity progressed as listed in the Table 1.

**Table 1:** Exercised training duration and intensity progress.

	Aerobic training (treadmill walking)	Strength training (maximal quadricep extension; velocity of 90°s^-1^)
Weeks 1–2	25 min at 60–70% VO_2peak_; 5 min 71–80% VO_2peak_	3 sets, 10 repetitions, 1 min rest between sets
Weeks 3–4	25 min at 60–70% VO_2peak_; 10 min 71–80% VO_2peak_	4 sets, 10 repetitions, 1 min rest between sets
Weeks 5–6	25 min at 60–70% VO_2peak_; 15 min 71–80% VO_2peak_	5 sets, 10 repetitions, 1 min rest between sets
Weeks 7–8	25 min at 60–70% VO_2peak_; 17 min 71–80% VO_2peak;_ 3 min 81–90% VO_2peak_	6 sets, 10 repetitions, 1 min rest between sets
Weeks 9–10	20 min at 60–70% VO_2peak_; 20 min 71–80% VO_2peak;_ 5 min 81–90% VO_2peak_	6 sets, 10 repetitions, 1 min rest between sets
Weeks 11–12	17 min at 60–70% VO_2peak_; 20 min 71–80% VO_2peak;_ 8 min 81–90% VO_2peak_	6 sets, 10 repetitions, 1 min rest between sets


All patients randomized to an exercise intervention received Digiwalker pedometers to serve as a motivational tool and to monitor daily physical activity. This training program is considered moderately intense, previously shown to increase aerobic capacity to ≥6 METs, a level sufficient to afford cardiorespiratory reserve (fitness) for performing normal activities of daily living (Langbein et al., 2002).

### Health Promotion Education

To minimize attention and educational bias, all patients received standard of care and attended a 12-session health promotion educational series provided by the same certified diabetes educator (CMM) consisting of various 30-min skill building sessions (Figure 1). During these visits, patients’ blood glucose levels from the previous 2 weeks were retrieved from study provided home glucometers (MediSense Precision Xtra). Initial assessments of personal lifestyle habits, including nutrition (food intake, nutritional value, shopping patterns, cooking/restaurant habits and food budget), body hygiene, eye, dental, foot care, and appropriate consultations needs, were determined. Medication management, blood glucose monitoring, graphing patients HbA_1c_ values over time and the interaction of these with food/fluid intake and daily activities were discussed. Patients access to the internet and current best source information on diabetes and related topics were demonstrated.

### Medical Management

Because physical activity lowers insulin resistance, blood glucose was monitored before and after each symptom-limited incremental treadmill stress test and exercise intervention session. Patients did not begin treadmill testing or exercise interventions until their blood glucose levels were ≥100 mg/dL but ≤300 mg/dL. All randomized patients performed and recorded daily blood glucose levels using study-provided test strips and calibrated glucometers throughout this study. Standard of care medications were employed for all subjects and included a combination of insulin, metformin, and sulfonylureas for glucose control and statins for anti-hyperlipidemic drugs. Adjusting a patient’s medication to better manage glycemic control was a priority health care issue and consistent with maintaining tight glycemic control. Comprehensive clinical management was maintained throughout the study by communicating treadmill stress test or exercise intervention results with the patient’s Primary Care Provider.

Cardiac performance of each randomized patient was visually monitored (leads II, V1, and V5) on a continuous basis. In the event the treadmill ECG was positive, patients were referred to cardiology for appropriate follow-up. Patients with indolent coronary artery disease, unstable angina, or other findings that made it unsafe to proceed with the exercise program were withdrawn from the study (Figure 2).

Blood pressure was determined by the auscultatory technique using a sphygmomanometer, a pre-gauged adult cuff, and a stethoscope. Ratings of perceived breathlessness, exertion, and right/left foot pain were recorded using Borg’s ratio scale.

All randomized patients received a detailed foot exam prior to, during, and after each exercise intervention session in conjunction with the bi-weekly Health Promotion classes. To minimize the risk of foot injury, all patients randomized to exercise intervention received and wore study-provided new well-fitting supportive athletic shoes.

### Nerve Conduction Studies

Limb temperatures were continuously monitored using an integrated surface temperature probe to minimize variation within and between patients. The temperature probe was placed distally on the leg at the dorsal surface of the ankle and distally on the volar surface of the forearm at the wrist crease. Prior to neurophysiologic evaluation, patient limbs were warmed (heating lamp) if the surface temperature was less than 32°C in the leg or less than 33°C at the wrist. In all cases, limb surface temperatures were maintained for all sequential nerve conduction studies.

To minimize inter-examiner variability and maximize neurophysiologic test/retest reliability, the same experienced neurologist (MAF) conducted all nerve conduction studies on days separate from all other testing activities (Dyck et al., 2013). A dedicated TECA Synergy electromyograph system was used for all nerve conduction studies. All nerve conduction studies were performed with standard protocols used in the Electromyography Laboratory at the Edward Hines Jr. Veterans Affairs Hospital. The patients dominant side was chosen. In patients with definable differences between the two sides, the side with the most prominent clinical findings was chosen. In all cases, the same limb was used for all three (baseline, 12-weeks, 24-weeks) conduction studies. A band pass filter setting of 20 Hz to 2 KHz was used with a sweep speed of 5 ms/div and a gain of 5 mV/div. The gain was increased, as needed, for low amplitude responses. Maximal responses were obtained using percutaneous electrical stimuli. Sensory nerve action potentials were recorded from sural (antidromic), median (antidromic to second digit), and ulnar nerves (antidromic to fifth digit). Distal motor nerve evoked compound muscle action potential (CMAP) potentials were recorded from tibial and peroneal nerves. F-waves were recorded as previously described (Fisher et al., 2007) and consisted of 20 separate recordings and minimal and mean recordable (>20 μV) F-wave latencies captured. Autonomic nerve function was not quantified in this study. *Normative Data Taskforce* electrodiagnostic reference values for select peripheral sensory and motor nerves for adult populations were used for comparison (Chen et al., 2016; Dillingham et al., 2016).

### Epidermal Nerve Fiber Density

At entry into this study, 12 patients volunteered to be biopsied at baseline, and again at 12-week post-intervention, for determination of epidermal nerve fiber density. Skin samples from the distal leg approximately 10 cm proximal to the lateral malleolus were obtained by punch biopsy (3 mm circular punch × 4 mm depth) and quantified for epidermal nerve fiber density (Therapath Neuropathology). Randomization of these 12 patients were as follows: sedentary controls (*n* = 4), aerobic exercise (*n* = 3), isokinetic strength training (*n* = 3), and combined intervention (*n* = 2). Post-intervention, three of these 12 patients (one sedentary control, two combined) declined to be biopsied a second time.

### Quantitative Sensory Testing

Quantitative sensory testing (QST) evaluation performed by the same examiner (ES) using a commercially available Computer Assisted Sensory Evaluator [CASE IV System] (WR Medical Electronics, Stillwater, MN, United States). A 4-2-1 methodology was employed. QST studies (vibration, cooling, heat-pain) were performed in a small dedicated quiet isolated enclosed testing room (24.4 ± 0.4°C) free from extraneous distractions and just prior to subjecting subject to symptom-limited treadmill testing. The same limb used for nerve conduction studies were used for QST evaluation. Foot temperature for all subjects was measured using a supplied infrared thermometer and rigorously maintained with the aid of a hair dryer or thermal sock at 33.4 ± 0.2°C.

### SF-36V Health Survey

The SF-36V health survey questionnaire was used to measure health-related quality of life (Kazis et al., 1998; Kazis et al., 2006). This survey is comprised of eight subscales and two overall component scores, all of which have demonstrated high-levels of internal consistency and discriminate validity when administered to groups of medically stable individuals. Patient aggregate responses for the eight distinct summary subscales and two component scores were compiled as a percentage of total points possible (Supplementary Table S1) using the RAND 36-item health survey table (Supplementary Table S2).

### Statistical Analyses

Data are presented either as the mean ± *SD* or as a box plot display showing the median, interquartile range along with the full range of data variation (min to max) of *N* observations unless noted otherwise. Statistical significance of parametric data within- or across-multiple experimental groups was determined by two-way ANOVA with a Tukey’s multiple comparison *post hoc* analysis. Statistical significance of parametric data involving two-groups was determined by Student’s *t*-test. Statistical significance of non-parametric data involving two-groups was determined by Mann–Whitney *U*-test whereas non-parametric data involving multiple-groups were analyzed by ANOVA on ranks (Kruskal–Wallis test). Variability between nerve conduction study primary outcome findings was observed within- and across-multiple experimental groups and is consistent with the presence of chronic severe length-dependent distal symmetric polyneuropathy within this diabetic patient population. Inherent limitations in statistical power were thus unavoidable and precluded an appropriately powered standard parametric statistical evaluation of these data. Statistical evaluation of individual patient electrophysiologic responses were subsequently determined using a *Fisher’s Exact* Test non-parametric assessment of within person changes. Improvement in nerve function was defined as >10% change from baseline of individual nerve conduction study responses. There is controversy over normative electrodiagnostic studies (Dillingham et al., 2016). In this study, a 10% change from baseline was chosen based on a large series of electrodiagnostic data accumulated from sixty centers (Bril et al., 1998). In all cases, *p* < 0.05 was considered statistically significant.

## Results

### Patient Sample and Demographics

A total of 1,079 patients were pre-screened at Edward Hines Jr. VA hospital for participation into this 24-week randomized controlled single-blinded study (Figure 1). Of the 1,079 patients pre-screened, 973 (90.2%) failed to meet the study inclusion criteria largely due to inability or unwillingness to commit to a 24-week study, poor glycemic control, physical limitations, history of alcohol/drug abuse or a range of clinical contraindications including cardiovascular, hepatic, pancreatic, renal, bladder, thyroid, vascular, autoimmune, hematological, neurological, respiratory pathologies. One hundred and six (9.8%) pre-screened patients satisfying the study inclusion criteria were enrolled and subsequently evaluated for indolent medical and physical endurance limitations. Sixty-one (58%) of the 106 enrolled patients were either medically ineligible (*n* = 40), were unable to commit to the study (*n* = 19), or exhibited a positive baseline treadmill stress test (*n* = 2) that required follow-up with cardiology (Figure 2). Forty-five (42%) of the 106 patients were medically cleared for entry into this study and were randomized to one of four experimental groups (Figure 2). Of these 45 randomized patients, 7 (15.6%) withdrew or were withdrawn from the study whereas 38 patients (84%) completed the study.

There were no statistical differences in age (*p* = 0.79), weight (*p* = 0.62), or BMI (*p* = 0.82) across the four experimental groups (Table 2). Twenty-six (57.8%) of the 45 patients were white, 16 (35.5%) were African American, and 3 (6.7%) were of Hispanic origin. Two-thirds or greater were chronic smokers and were well represented across all experimental groups (Table 2). Non-fasting blood glucose levels across experimental groups at baseline was 177.0 ± 6.8 mg/dL whereas the average HbA_1c_ levels across groups at baseline ranged from 7.2 to 8.0% and remained stable throughout the course of this 24-week study (Table 2). Independent of intervention, patients maintained their overall body weight or body mass (Table 2) and glycemic control throughout this study (Table 2), suggesting that any exercise-induced metabolic bias elicited by the aerobic-, isokinetic strength-, or combined exercise protocols was most likely minimal.

**Table 2:** Baseline patient demographics and clinical laboratory profiles.

Characteristics	Sedentary	Aerobic	Strength	Aerobic + strength	*p*
Age (years)	61.0 ± 7.0 (12)	61.9 ± 8.3 (11)	64.2 ± 9.5 (11)	63.0 ± 6.6 (11)	0.79
Height (cm)	178.1 ± 5.8 (12)	181.0 ± 8.6 (11)	179.9 ± 5.3 (11)	177.4 ± 7.1 (11)	0.59
**Weight (kg)**					
Baseline	104.7 ± 14.4 (12)	109.9 ± 19.7 (11)	102.6 ± 16.3 (11)	101.3 ± 14.4 (11)	0.62
Intervention	103.7 ± 12.9 (11)	109.8 ± 18.5 (11)	102.9 ± 17.0 (11)	97.1 ± 9.7 (9)	0.33
*p*	0.98	1.00	1.00	0.72	
12-week post	100.5 ± 10.5 (9)	109.8 ± 18.3 (11)	103.8 ± 17.6 (10)	98.4 ± 10.7 (8)	0.38
*p*	0.85	1.00	0.99	0.97	
**BMI (kg/m^2^)**					
Baseline	32.9 ± 3.5 (12)	33.4 ± 5.4 (11)	31.5 ± 4.1 (11)	32.4 ± 6.3 (11)	0.82
Intervention	32.3 ± 2.8 (11)	33.4 ± 5.0 (11)	31.6 ± 4.2 (11)	30.8 ± 4.0 (9)	0.53
*p*	0.88	1.00	1.00	0.76	
12-week post	31.5 ± 2.6 (9)	33.4 ± 4.9 (11)	31.9 ± 4.3 (9)	31.5 ± 3.8 (8)	0.68
*p*	0.83	1.00	0.99	0.96	
**Gender**					
Male	12	10	11	10	
Female	0	1	0	1	
**Ethnicity**					
Caucasian	6 (50%)	7 (64%)	6 (54%)	7 (64%)	
African American	6 (50%)	3 (27%)	4 (36%)	3 (27%)	
Hispanic	0 (0%)	1 (9%)	1 (9%)	1 (9%)	
No. smokers (%)	8 (67%)	9 (82%)	8 (73%)	10 (91%)	
Pk-years	14.2 ± 21.2 (12)	32.2 ± 40.7 (11)	10.6 ± 5.3 (11)	27.5 ± 21.1 (11)	0.15
DM (years)	12.6 ± 11.1 (12)	10.9 ± 4.1 (11)	10.1 ± 6.7 (11)	8.9 ± 6.3 (11)	0.70
**Blood glucose (mg/dL)**					
Pre-TM test	157.3 ± 49.5 (12)	188.6 ± 65.6 (11)	181.5 ± 48.9 (11)	180.7 ± 43.2 (11)	0.51
Post-TM test	129.3 ± 41.7 (12)	169.4 ± 46.0 (11)	138.5 ± 48.7 (11)	149.5 ± 46.1 (11)	0.20
*p*	0.15	0.44	0.05	0.12	
**HbA1C (percent)**					
Baseline	7.8 ± 0.5 (12)	8.0 ± 1.0 (11)	8.0 ± 1.0 (11)	7.2 ± 0.7 (11)	0.09
Intervention	7.7 ± 1.0 (12)	8.0 ± 1.5 (11)	7.4 ± 1.0 (11)	7.3 ± 1.0 (9)	0.51
*p*	0.96	1.00	0.40	0.96	
12-week post	7.2 ± 1.1 (9)	7.9 ± 1.3 (11)	8.0 ± 1.2 (10)	7.1 ± 0.7 (9)	0.19
*p*	0.42	0.98	0.41	0.86	
**Triglycerides (mg/dL)**	157.7 ± 84.5 (12)	125.0 ± 57.1 (11)	144.5 ± 68.2 (11)	162.0 ± 60.0 (11)	0.59
Cholesterol (mg/dL)	139.3 ± 25.1 (12)	156.4 ± 36.2 (11)	158.5 ± 39.7 (11)	159.9 ± 48.7 (11)	0.53
HDL-cholesterol (mg/dL)	41.4 ± 29.0 (12)	42.8 ± 13.5 (11)	40.7 ± 14.0 (11)	38.4 ± 4.8 (11)	0.95
LDL-cholesterol (mg/dL)	68.4 ± 23.3 (12)	88.6 ± 31.2 (11)	89.0 ± 42.5 (11)	89.2 ± 48.0 (11)	0.45
**Creatinine (mg/dL)**	1.1 ± 0.2 (12)	1.0 ± 0.1 (11)	0.9 ± 0.1 (11)	1.0 ± 0.2 (11)	0.04
BUN (mg/dL)	17.0 ± 5.5 (12)	19.0 ± 5.9 (11)	16.5 ± 6.7 (11)	17.8 ± 5.6 (11)	0.77
AST (units/l)	20.8 ± 5.6 (12)	13.5 ± 6.3 (11)	19.5 ± 9.6 (11)	23.6 ± 15.2 (11)	0.12
TSH (μIU/ml)	1.3 ± 0.6 (12)	1.8 ± 0.9 (11)	2.0 ± 1.6 (11)	1.8 ± 0.7 (11)	0.42


*Data shown are the means ± SD of (*N*) patients. For gender, ethnicity, and smokers, the number of patients per group is shown. Where applicable, data are analyzed by one-way ANOVA with Tukey’s multiple comparison analysis. Non-fasting blood glucose levels were determined at rest prior to symptom-limited treadmill testing (Pre-TM test) and immediately following treadmill testing (post-TM test). For within group analyses, baseline values are compared with intervention; intervention values are compared with 12-week post-intervention values.*

### Effect of Exercise Training on Self-Reported Health Status

**FIGURE 3:**
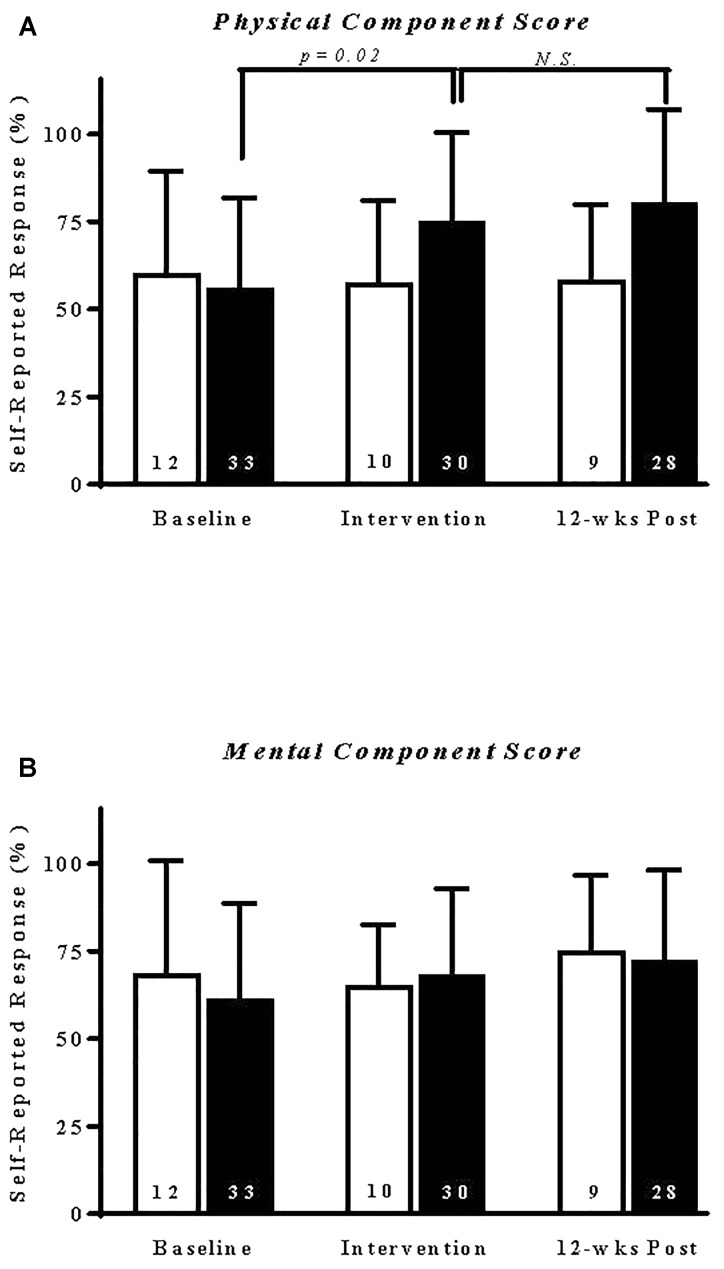
Exercise selectively elicits a sustained increase in SF-36V patient self-reported physical component score. Data shown are the means ± SD of self-reported responses from patients randomized to sedentary control **(open bars)** or combined exercise **(solid bars)** experimental groups. **(A)** Physical or **(B)** mental component scores are expressed as a percentage of weighted score (Supplementary Table S2) determined at entry into the study (baseline), immediately following intervention, and again at 12-week post-intervention of *N* responding patients, as indicated. Non-parametric data were analyzed by ANOVA on ranks (Kruskal–Wallis test). *N.S.*, not significant. Note that one patient randomized to exercise did not complete the 12-week post SF-36V questionnaire.

Patients randomized to exercise training reported a significant (*p* = 0.02) and sustained improvement in their physical component scores (Figure 3A). By comparison, exercise intervention did not alter patient mental component scores (Figure 3B). Similarly, exercise had no immediate or sustained effect on individual SF-36V subscale performance measures (Supplementary Figure S1 and Supplementary Table S3). A comparison within and across study groups of the specific effects of exercise type on health status component scores is shown in Table 3. A 12-week course of aerobic exercise elicited a statistically significant (*p* = 0.01) and sustained improvement, compared to baseline values, in physical component scores. In contrast, patients receiving isokinetic strength or the combination of aerobic–isokinetic strength training reported no significant changes over baseline in perceived physical or mental component health scores (Table 3).

**Table 3:** Relative effect of exercise type on self-reported health component scores using the SF-36V questionnaire.

Scale	Sedentary	Aerobic	Strength	Aerobic + strength	*p*
**Physical component score**					
Baseline	60.4 ± 29.1 (12)	52.3 ± 26.1 (11)	56.8 ± 25.2 (11)	59.1 ± 28.0 (11)	0.96
Intervention	57.5 ± 23.7 (10)	86.4 ± 17.2 (11)	65.0 ± 26.9 (10)	72.2 ± 29.2 (9)	0.06
*p*	1.00	0.01	1.00	0.98	
12-week post	58.3 ± 21.7 (9)	86.4 ± 17.2 (11)	77.5 ± 32.2 (10)	75.0 ± 32.3 (7)	0.14
*p*	1.00	1.00	0.66	1.00	
**Mental component score**					
Baseline	68.8 ± 32.2 (12)	63.6 ± 30.3 (11)	59.1 ± 25.7 (11)	61.4 ± 28.2 (11)	0.81
Intervention	65.0 ± 17.5 (10)	72.7 ± 20.8 (11)	60.0 ± 26.9 (10)	72.2 ± 26.4 (9)	0.60
*p*	1.00	1.00	1.00	1.00	
12-week post	75.0 ± 21.7 (9)	75.0 ± 19.4 (11)	67.5 ± 33.4 (10)	75.0 ± 25.0 (7)	0.99
*p*	1.00	1.00	1.00	1.00	


*Data shown are the means ± SD of (*N*) patients. These non-parametric data were analyzed using a Kruskal–Wallis test with Dunn’s multiple comparison analysis. For within group analyses, baseline values are compared with intervention; intervention values are compared with 12-week post-intervention values.*

### Effect of Exercise Training on Treadmill Endurance and Maximally Achieved Metabolic Parameters

**FIGURE 4:**
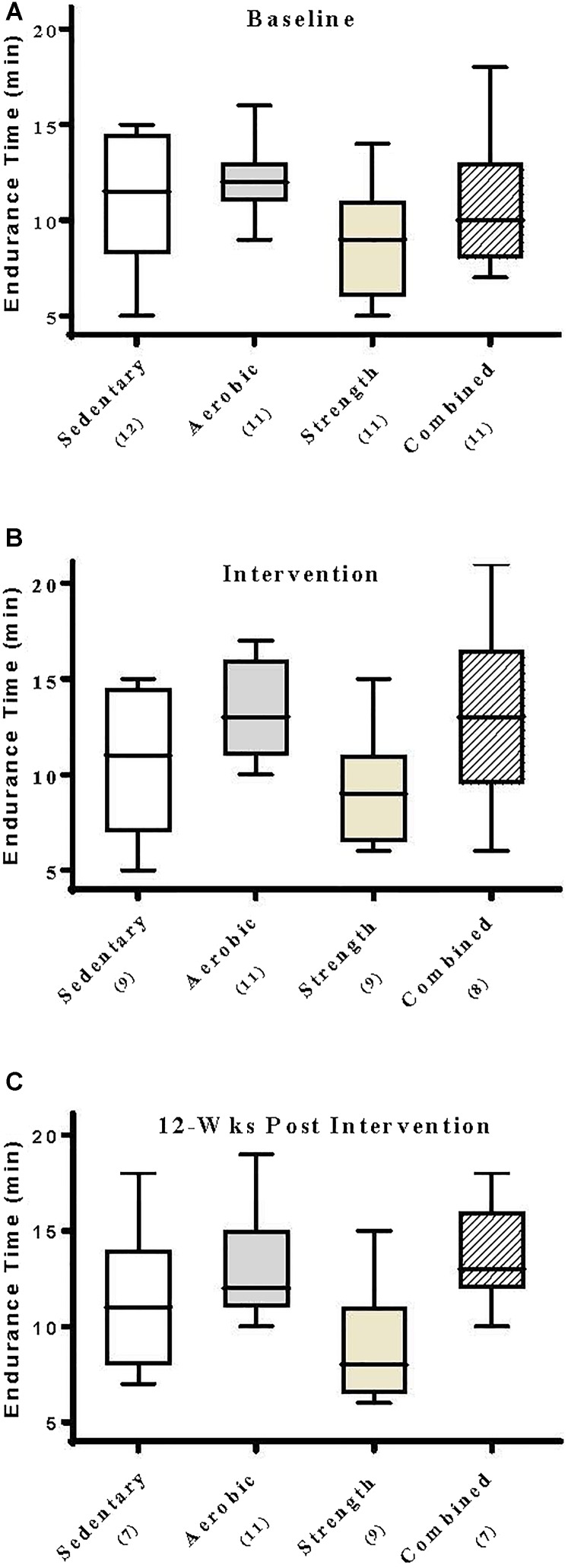
Aerobic exercise selectively elicits improved treadmill endurance times. Data shown are the median, interquartile range, and minimum–maximum range of patient treadmill endurance times at **(A)** entry into the study (baseline), **(B)** immediately following intervention, and again at **(C)** 12-week post-intervention of *N* responding patients, as indicated. Parametric data were analyzed by two-way ANOVA with Tukey’s multiple comparison *post hoc* analysis. At baseline, all groups were statistically indistinguishable. Patients that had received aerobic training exhibited a strong statistical trend (*p* = 0.07) toward prolonged treadmill endurance times that was sustained (*p* = 0.02) when compared to those patients that had received strength training alone.

At entry into this study, patients undergoing a symptom-limited modified Bruce treadmill test exhibited endurance times that were statistically indistinguishable across experimental groups (*p* = 0.14, range 9–12 min, Figure 4A). By comparison, those patients whom had undergone a 12-week intervention regimen of aerobic training or a 12-week combined aerobic–isokinetic strength training regimen exhibited modestly improved treadmill endurance times that were sustained throughout the 12-week post-training period (Figure 4B,C). In contrast, patients whom had undergone isokinetic strength training did not exhibit improved endurance times (Figure 4). Compared within and across experimental groups, subjecting patients to aerobic-, isokinetic strength-, or the combined intervention had no immediate or sustained effect on maximally achieved (peak) VO_2_ observed during subsequent treadmill testing evaluations (Table 4). Collectively, these findings would suggest that the modest changes observed in treadmill endurance times by aerobically trained subjects was most likely unrelated to changes in metabolic conditioning.

**Table 4:** Relative effect of exercise type on peak metabolic parameters.

Characteristics	Sedentary	Aerobic	Strength	Aerobic + strength	*p*
**Peak VE (l/min)**					
Baseline	62.9 ± 15.3 (12)	63.5 ± 18.8 (11)	54.3 ± 12.9 (11)	62.0 ± 13.3 (11)	0.46
Intervention	59.9 ± 10.6 (9)	67.0 ± 22.2 (11)	60.6 ± 12.1 (9)	65.0 ± 14.3 (8)	0.72
*p*	0.87	0.93	0.51	0.89	
12-week post	57.4 ± 13.1 (7)	73.2 ± 27.3 (11)	56.4 ± 12.4 (9)	64.9 ± 15.8 (7)	0.22
*p*	0.93	0.80	0.76	1.00	
**Peak VO_2_ (ml.kg^-1^.min^-1^)**					
Baseline	17.4 ± 3.9 (12)	18.9 ± 4.2 (11)	16.5 ± 3.8 (11)	18.9 ± 3.6 (11)	0.39
Intervention	17.0 ± 4.6 (9)	18.7 ± 5.1 (11)	17.2 ± 3.0 (9)	19.5 ± 4.5 (8)	0.59
*p*	0.98	0.99	0.87	0.95	
12-week post	18.2 ± 4.6 (7)	19.3 ± 5.3 (11)	16.5 ± 2.2 (9)	20.6 ± 4.4 (7)	0.29
*p*	0.85	0.96	0.88	0.86	
**Peak RER (VCO_2_/VO_2_)**					
Baseline	1.2 ± 0.1 (12)	1.2 ± 0.2 (11)	1.1 ± 0.1 (11)	1.1 ± 0.1 (11)	0.11
Intervention	1.2 ± 0.1 (9)	1.2 ± 0.1 (11)	1.2 ± 0.1 (9)	1.2 ± 0.1 (8)	0.41
p	0.97	1.00	0.09	0.05	
12-week post	1.2 ± 0.1 (7)	1.2 ± 0.1 (11)	1.1 ± 0.1 (9)	1.1 ± 0.0 (7)	0.03
*p*	0.98	1.00	0.11	0.08	
**Peak METS**					
Baseline	5.0 ± 1.1 (12)	5.4 ± 1.2 (11)	4.7 ± 1.1 (11)	5.4 ± 1.0 (11)	0.38
Intervention	4.9 ± 1.3 (9)	5.4 ± 1.4 (11)	4.9 ± 0.9 (9)	5.6 ± 1.3 (8)	0.55
*p*	0.98	1.00	0.88	0.93	
12-week post	5.2 ± 1.3 (7)	5.5 ± 1.5 (11)	4.8 ± 0.6 (9)	5.9 ± 1.2 (7)	0.37
*p*	0.88	0.98	0.97	0.87	


*Data shown are the means ± SD of (*N*) patients. Data are analyzed by one-way ANOVA with Tukey’s multiple comparison analysis. For within group analyses, baseline values are compared with intervention; intervention values are compared with 12-week post-intervention values.*

### Effect of Exercise Training on Peripheral Sensory and Motor Nerve Electrodiagnostic Findings

At entry into this study, patients exhibited marked deficits in sensory nerve electrodiagnostic findings, consistent with chronic (>10 years) diabetes. Of the 45 patients randomized, 37 (82%) had absent sural nerve responses, 19 (42%) had absent median sensory nerve responses, and 17 (38%) had absent ulnar sensory nerve responses. By comparison, responses from tibial nerves were absent in only three (7%) subjects while responses from peroneal nerves were absent in five (11%) patients.

**FIGURE 5:**
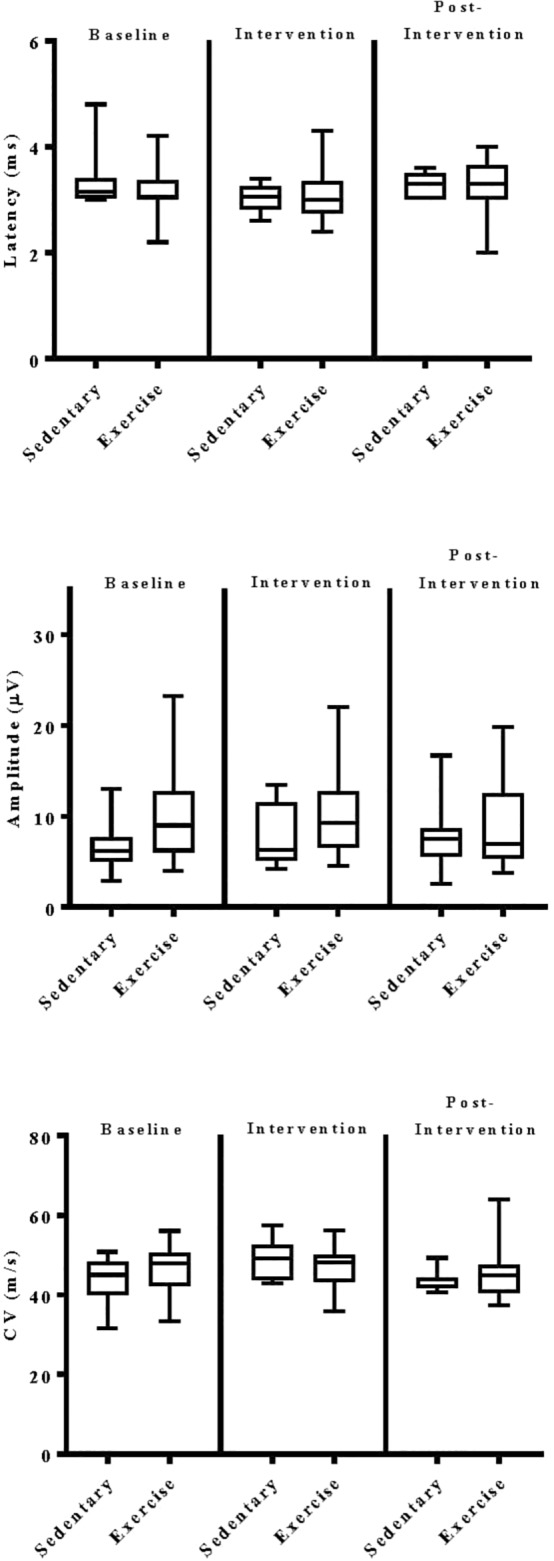
Group analyses of the effect of exercise on ulnar sensory nerve evoked electrodiagnostic parameters. Data shown are the median, interquartile range, and minimum–maximum range of latency, amplitude, and calculated conduction velocity elicited from antidromic stimulation of ulnar sensory nerves at entry into the study (baseline), immediately following intervention, and again at 12-week post-intervention of responding patients, as indicated. Parametric data were analyzed by two-way ANOVA with Tukey’s multiple comparison *post hoc* analysis. The total number of patients with measurable responses at baseline (sedentary controls, *N* = 8; exercise, *N* = 20), immediately following intervention (sedentary controls, *N* = 6; exercise, *N* = 16), and at 12-week post-intervention (sedentary controls, *N* = 7; exercise, *N* = 21) were group analyzed. At all three time points evaluated, there was no significant differences observed within and across experimental groups.

**FIGURE 6:**
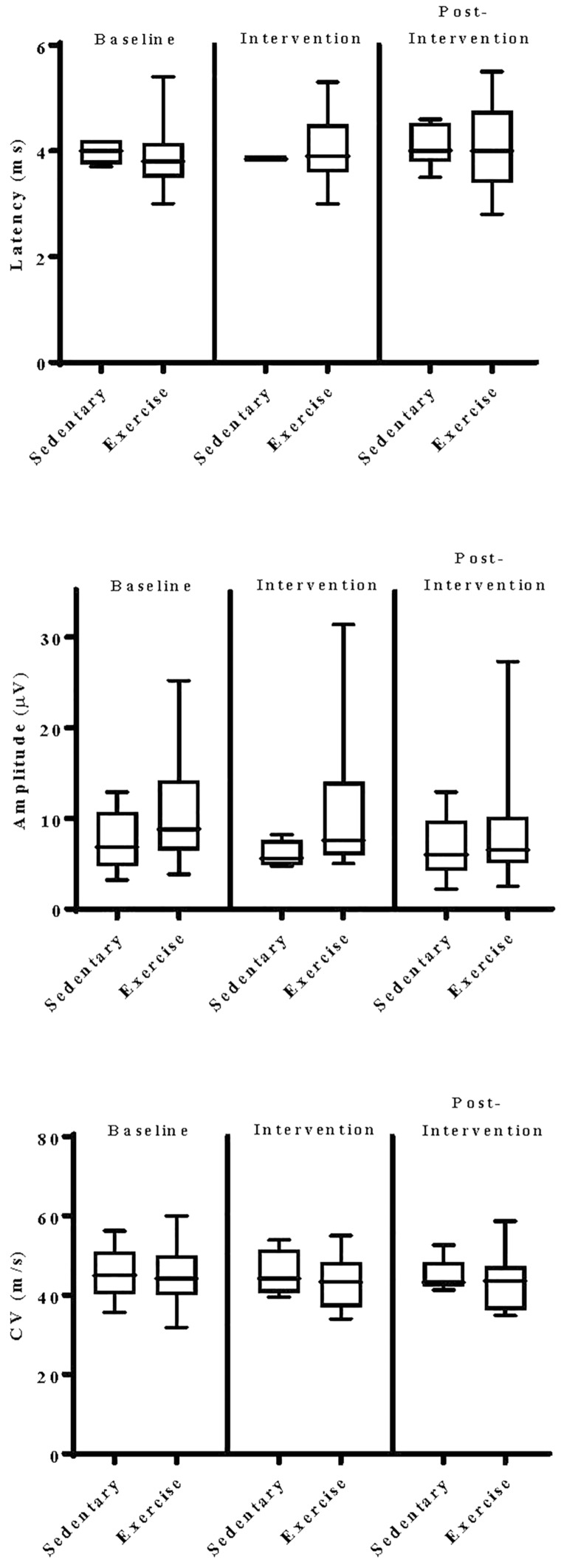
Group analyses of the effect of exercise on median sensory nerve evoked electrodiagnostic parameters. Data shown are the median, interquartile range, and minimum–maximum range of latency, amplitude, and calculated conduction velocity elicited from antidromic stimulation of median sensory nerves at entry into the study (baseline), immediately following intervention, and again at 12-week post-intervention of responding patients, as indicated. Parametric data were analyzed by two-way ANOVA with Tukey’s multiple comparison *post hoc* analysis. The total number of patients with measurable responses at baseline (sedentary controls, *N* = *5*; exercise, *N* = *21*), immediately following intervention (sedentary controls, *N* = *4*; exercise, *N* = *18*), and at 12-week post-intervention (sedentary controls, *N* = *6*; exercise, *N* = *17*) were group analyzed. At all three time points evaluated, there was no significant differences observed within and across experimental groups.

**FIGURE 7:**
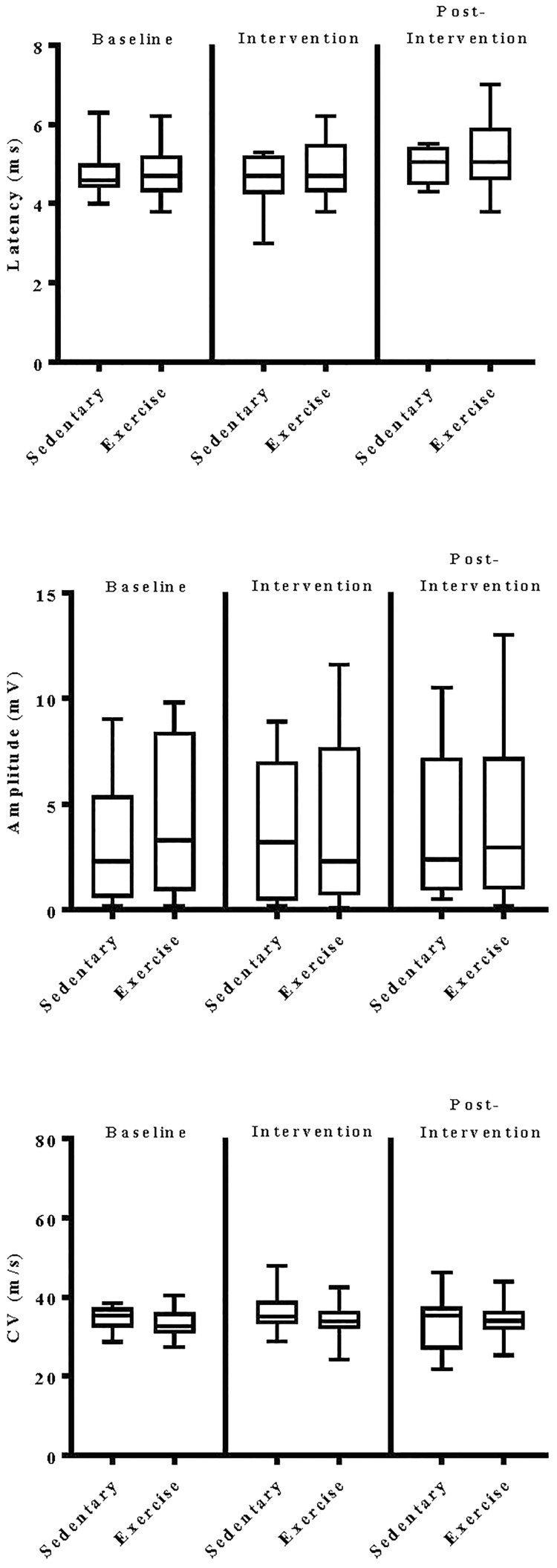
Group analyses of the effect of exercise on tibial nerve evoked electrodiagnostic parameters. Data shown are the median, interquartile range, and minimum–maximum range of latency, amplitude, and calculated conduction velocity elicited from antidromic stimulation of tibial nerves at entry into the study (baseline), immediately following intervention, and again at 12-week post-intervention of responding patients, as indicated. Parametric data were analyzed by two-way ANOVA with Tukey’s multiple comparison *post hoc* analysis. The total number of patients with measurable responses at baseline (sedentary controls, *N* = *11*; exercise, *N* = *31*), immediately following intervention (sedentary controls, *N* = *9*; exercise, *N* = *27*), and at 12-week post-intervention (sedentary controls, *N* = *8*; exercise, *N* = *26*) were group analyzed. At all three time points evaluated, there was no significant differences observed within and across experimental groups.

**FIGURE 8:**
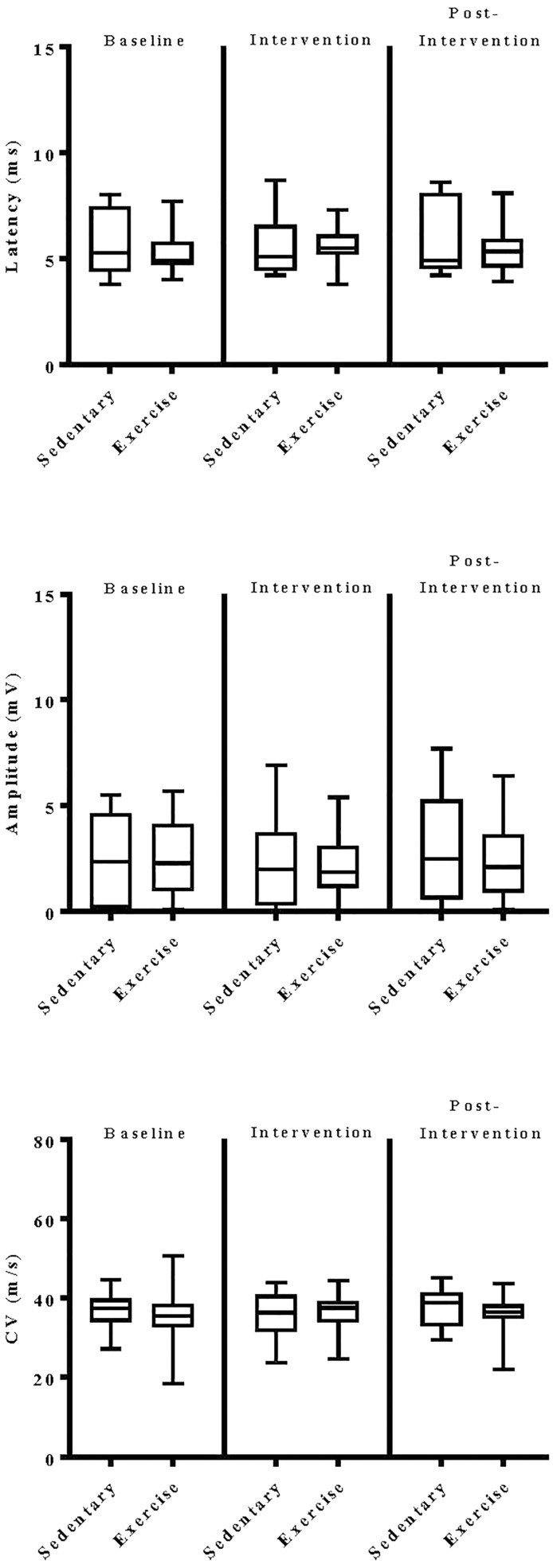
Group analyses of the effect of exercise on peroneal nerve evoked electrodiagnostic parameters. Data shown are the median, interquartile range, and minimum–maximum range of latency, amplitude, and calculated conduction velocity elicited from antidromic stimulation of peroneal nerves at entry into the study (baseline), immediately following intervention, and again at 12-week post-intervention of responding patients, as indicated. Parametric data were analyzed by two-way ANOVA with Tukey’s multiple comparison *post hoc* analysis. The total number of patients with measurable responses at baseline (sedentary controls, *N* = *10*; exercise, *N* = *30*), immediately following intervention (sedentary controls, *N* = *8*; exercise, *N* = *26*), and at 12-week post-intervention (sedentary controls, *N* = *7*; exercise, *N* = *26*) were group analyzed. At all three time points evaluated, there was no significant differences observed within and across experimental groups.

The impact of exercise on electrodiagnostic findings within and across experimental groups was evaluated using both parametric and non-parametric statistical approaches. When analyzed parametrically, exercise, regardless of type, did not appear to significantly alter sensory or motor nerve electrodiagnostic findings among those patients exhibiting measurable responses (Figure 5–8). Similarly, F-waves were unaffected by exercise intervention (data not shown). Whereas exercise may not have elicited a statistically significant improvement in electrodiagnostic-assessed nerve function, these data demonstrate that exercise, regardless of type, does not exacerbate peripheral sensory or motor nerve injury in diabetic neuropathic patients (Figure 5–8).

Using a *Fisher’s Exact* Test analysis of these composite electrodiagnostic findings, we observed a modest statistically significant (*p* = 0.01) beneficial effect of exercise on sensory nerve function (Table 5A). Importantly, the beneficial effect of exercise on sensory nerve function was enhanced (*p* = 0.03) during the post-intervention interval (Table 5A), possibly due to a delayed physiologic response. Qualitative improvement in sensory electrodiagnostic studies included the presence of sural sensory nerve action potentials that had been absent in the prior baseline evaluation. In contrast, patients whom had undergone exercise intervention did not exhibit statistically significant (*p* = 0.71) improvement in motor nerve composite electrodiagnostic findings (Table 5B).

**Table 5A:** *Fisher’s Exact* Test contingency table of composite electrodiagnostic findings (latency, amplitude, CV) elicited from sural, ulnar, and median sensory nerves.

	Improved	Not improved	*p*
**Sedentary**			
Baseline vs. 12-week intervention	20	70	
**Exercise**			
Baseline vs. 12-week intervention	29	240	0.01
Baseline vs. post-intervention	47	216	0.03


*Data shown are the number of individual patients exhibiting improvement of individual responses in sensory nerve latency (>10% decrease), amplitude (>10% increase), or conduction velocity (>10% increase) over baseline electrodiagnostic findings obtained upon entry into this study.*

**Table 5B:** *Fisher’s Exact* Test contingency table of composite electrodiagnostic findings (latency, amplitude, CV) elicited from tibial and peroneal nerves.

	Improved	Not improved	*p*
**Sedentary**			
Baseline vs. 12-week intervention	13	36	
**Exercise**			
Baseline vs. 12-week intervention	38	123	0.71


*Data shown are the number of individual patients exhibiting improvement of individual responses in motor nerve latency (>10% decrease), amplitude (>10% increase), or conduction velocity (>10% increase) over baseline electrodiagnostic findings obtained upon entry into this study.*

### Effect of Exercise Training on Epidermal Nerve Fiber Density

At entry into this study, 11 (92%) of the 12 patients that volunteered to be biopsied exhibited abnormal epidermal nerve fiber density levels (<5.4). Three of the six patients (50%) that had undergone exercise intervention exhibited a marked 1.9 ± 0.3-fold improvement in epidermal nerve fiber density with a fourth patient exhibiting no change in epidermal nerve fiber density. By comparison, two of the three sedentary patients who underwent repeat testing showed a progressive loss of epidermal nerve fiber density with a third having no detectable levels of epidermal nerve fiber density at baseline.

### Effect of Exercise Training on Quantitative Sensory Testing

Marked small-fiber neurologic deficits within this patient population were also evident from quantitative sensory testing studies. At entry into this study, patient responses to vibration stimuli were nearly negligible (Table 6). By comparison, patient responses to a cooling sensation were varied (range, 13–21 JND) whereas responses to the onset of heat pain were delayed (range 19–20 JND). Exercise intervention, regardless of type, did not significantly alter patient perception of vibration, cooling, or heat-pain (Table 6).

**Table 6:** Relative effects of exercise on quantitative sensory testing.

Scale	Sedentary	Aerobic	Strength	Aerobic + strength	*p*
**Vibration threshold (JND)**					
Baseline	23.1 ± 1.6 (12)	24.0 ± 1.2 (11)	23.4 ± 1.6 (11)	22.5 ± 3.5 (11)	0.44
Intervention	21.8 ± 2.5 (10)	24.2 ± 1.3 (11)	23.3 ± 1.6 (10)	22.7 ± 3.5 (8)	0.13
*p*	0.32	0.95	0.99	0.99	
12-week post	22.8 ± 2.1 (9)	23.3 ± 2.0 (11)	23.2 ± 1.3 (10)	23.5 ± 1.8 (8)	0.88
*p*	0.55	0.37	0.99	0.86	
**Cooling threshold (JND)**					
Baseline	14.0 ± 4.7 (12)	20.8 ± 5.1 (11)	13.4 ± 5.0 (11)	16.4 ± 5.5 (11)	0.01
Intervention	16.7 ± 4.9 (10)	20.5 ± 4.1 (11)	14.2 ± 6.5 (10)	17.6 ± 4.3 (8)	0.06
*p*	0.33	0.99	0.95	0.85	
12-week post	17.1 ± 3.0 (9)	20.8 ± 4.5 (11)	14.1 ± 6.2 (10)	18.4 ± 3.6 (8)	0.02
*p*	0.98	0.99	1.00	0.94	
**Heat pain onset threshold (JND)**					
Baseline	19.6 ± 2.9 (12)	19.8 ± 3.0 (11)	20.3 ± 1.9 (11)	19.9 ± 2.3 (11)	0.93
Intervention	19.3 ± 3.2 (10)	20.3 ± 1.9 (11)	20.9 ± 1.4 (10)	20.5 ± 2.1 (8)	0.45
*p*	0.97	0.85	0.68	0.84	
12-week post	20.2 ± 3.2 (9)	19.5 ± 1.3 (11)	20.3 ± 1.5 (10)	19.8 ± 2.5 (8)	0.83
*p*	0.80	0.67	0.69	0.82	
**Heat pain half-maximal threshold (JND)**					
Baseline	22.6 ± 3.6 (12)	23.5 ± 2.6 (11)	23.0 ± 1.5 (11)	22.6 ± 2.7 (11)	0.84
Intervention	23.5 ± 3.8 (10)	23.6 ± 2.6 (11)	23.7 ± 1.8 (10)	23.3 ± 3.0 (8)	0.99
*p*	0.84	1.00	0.61	0.86	
12-week post	23.6 ± 3.9 (9)	22.8 ± 2.4 (11)	23.5 ± 1.7 (10)	22.9 ± 3.0 (8)	0.89
*p*	1.00	0.74	0.96	0.96	
**Heat pain sensitivity (JND)**					
Baseline	3.2 ± 1.3 (12)	3.7 ± 2.2 (11)	2.8 ± 1.3 (11)	2.7 ± 1.3 (11)	0.44
Intervention	4.3 ± 2.1 (10)	3.3 ± 1.7 (11)	2.8 ± 1.1 (10)	2.9 ± 1.5 (8)	0.19
*p*	0.28	0.89	1.00	0.95	
12-week post	3.3 ± 1.5 (9)	3.3 ± 2.1 (11)	3.2 ± 1.1 (10)	3.1 ± 1.3 (8)	0.99
*p*	0.40	1.00	0.73	0.95	


*Data shown are the means ± SD of just noticeable differences (JND) from (*N*) patients. Data are analyzed by one-way ANOVA with Tukey’s multiple comparison analysis. For within group analyses, baseline values are compared with intervention; intervention values are compared with 12-week post-intervention values.*

## Discussion

Forty-five subjects with long-standing type 2 diabetes mellitus and length-dependent distal symmetric polyneuropathy were randomized to a 24-week clinical trial conducted to determine whether a structured aerobic-, isokinetic strength-, or combined aerobic–isokinetic strength exercise intervention program, compared with sedentary controls, would alter peripheral nerve function. Exercise, regardless of type, neither improved or impaired sensory or motor nerve electrodiagnostic findings across or within groups when analyzed parametrically. Non-parametric analyses of composite electrodiagnostic findings of individual responses supported a modest beneficial effect of exercise, however, on sensory nerve function. Importantly, the beneficial effect of exercise on sensory nerve function was even more evident following the intervening post-intervention interval. In contrast, exercise did not elicit any detectable benefit to tibial or peroneal motor nerves. Eleven (92%) of 12 patients that had volunteered to be biopsied exhibited abnormal levels of epidermal nerve fiber densities. Three of six patients that had undergone exercise intervention exhibited a marked improvement in epidermal nerve fiber density. By comparison, none of three sedentary patients whom agreed to be biopsied a second time showed improvement in epidermal nerve fiber density. Compared to baseline values within groups, and compared with sedentary values across groups, neither aerobic-, isokinetic strength-, or the combination of aerobic–isokinetic strength exercise intervention significantly altered metabolic or hemodynamic findings in this chronic diabetic patient population. Patients that underwent aerobic- or the combined aerobic–isokinetic strength exercise intervention demonstrated an increase in treadmill test duration that was sustained over the 12-week post-intervention period. Aerobic exercise intervention alone elicited a significant and sustained improvement in overall perceived SF-36V physical, but not mental, component score.

While many clinical trials (Pan et al., 1997; Tuomilehto et al., 2001; Knowler et al., 2002; Lindström et al., 2006; Ramachandran et al., 2006; Li et al., 2008; Diabetes Prevention Program Research Group et al., 2009; Colberg et al., 2010a,b, 2016; American Diabetes Association, 2014) offer support and guidance for those *at risk* for developing diabetes (prediabetes), it is less clear whether structured lifestyle intervention programs will also prove safe and clinically effective at slowing the progression of already established disease as well as altering the progression of associated retinopathic, nephropathic, or neuropathic complications. Previous studies suggest that the beneficial effects of structured exercise intervention programs on diabetes management are primarily realized through improved insulin sensitivity and blood glucose control (Boule et al., 2001; Dunstan et al., 2002; Castaneda et al., 2002; Sigal et al., 2004; Cauza et al., 2005; Church et al., 2010). Improved glycemic control, leading to lower HbA_1_c levels, is also associated with reduced onset and progression of microvascular (retinopathy and nephropathy) and neuropathic complications (Diabetes Control and Complications Trial Research Group et al., 1993, Diabetes Control and Complications Trial/Epidemiology of Diabetes Interventions and Complications Research Group et al., 2000; Stratton et al., 2000; Martin et al., 2006; ADVANCE Collaborative Group et al., 2008; Duckworth et al., 2009; Ismail-Beigi et al., 2010).

In our experience, a 12-week-on 12-week-off course of exercise, regardless of type, did not significantly alter HbA_1_c levels in well-managed glycemic controlled patients with long-standing diabetes. Given that HbA_1_c levels reflect an average glycemia over several months (Sacks et al., 2011), marked exercise-dependent changes in HbA_1_c levels over the 24-week course of this study were neither anticipated or observed. Immediately following a symptom-limited treadmill challenge, however, non-fasting blood glucose levels in all patients fell between 13 and 20%. The acute effect of exercise on glycemic control was not, however, sustained by either a 12-week course of structured exercise or following a 12-week post-intervention period. The observed effect of acute exercise on blood glucose levels was therefore transient and most consistent with a rapid short-lived skeletal muscle dependent GLUT4-mediated mechanism of glycemic control (Stanford and Goodyear, 2014). While short-term moderately intense structured exercise programs, such as the ones used in this study, do little to sustain acute-exercise induced effects on glycemic control, more extensive and prolonged structured programs of physical activity may be necessary to even modestly alter the progression of diabetes in patients with established disease (Gregg et al., 2012). This, however, may not necessarily be the case for diabetes associated vascular complications.

Beyond glycemic control, structured exercise intervention programs are thought to improve vascular endothelial function by increasing endothelial-derived nitric oxide (NO) bioavailability (Green et al., 2004) along with decreasing the burden of localized oxidative stress (Banerjee et al., 2003). Early studies have shown that a short-term (4-week) program of physical activity increases agonist-induced endothelium-dependent vasodilatory capacity and average peak flow velocity in patients with stable coronary artery disease (Hambrecht et al., 2003), suggesting that the vascular endothelium is itself a highly adaptable regulatory organ that is quite responsive to lifestyle intervention programs (DeSouza et al., 2000). Endothelial dysfunction culminating in macro- and microvascular damage plays a key role in the initiation of diabetes associated complications. Peripheral nerves of long-standing diabetic patients show extensive microvascular pathology, including basement membrane thickening, loss of pericyte investment, as well as endoneurial endothelial cell hyperplasia, which strongly correlates clinically with nerve function deficits. Secondary to reduced nerve blood flow and increased endoneurial vascular resistance, ischemia and hypoxia are considered the two most prevailing pathophysiological mechanisms by which chronic diabetes alters peripheral nerve function (Nakuda, 2014). Given that exercise improves vascular endothelial function, we speculated that an increase in endoneurial blood flow elicited by exercise may afford a measure of protection against further ischemic insult to affected peripheral nerve fibers.

All subjects randomized to this study had clinical and electrodiagnostic findings consistent with length-dependent distal symmetric polyneuropathy. Sensory nerve dysfunction was particularly evident in this limited patient population, as sural nerve electrodiagnostic responses were absent in 82% of study subjects while 42% had absent median- and 38% had absent ulnar-sensory nerve responses. Marked small-fiber neurologic deficits within this patient population were evident from quantitative sensory testing studies. Similarly, eleven out of 12 (92%) subjects tested also had abnormal epidermal nerve fiber densities, consistent with severe small-fiber neuropathy. In comparison to sensory nerve fibers, motor nerve fibers examined were not as affected, which perhaps contributed to the relatively positive quality of life measures indicated at baseline and throughout this study on the standardized SF-36V health survey questionnaire.

In those patients where electrodiagnostic responses were initially measurable, exercise, regardless of type, had no statistically significant beneficial or detrimental effects on motor nerve electrodiagnostic findings nor on sensory nerve studies as parametrically analyzed within and across experimental groups. Similarly, exercise intervention did not significantly alter patient perceptions of vibration, cooling, or heat-pain as determined by quantitative sensory testing. Aerobic exercise did, however, improve subject treadmill endurance times as well as overall physical component scores, supporting a modest clinical benefit for aerobic exercise in the management of the diabetic neuropathic patient. While these electrodiagnostic findings and quality of life performance measures would indicate that short-term exercise intervention programs such as the ones used in this study are largely ineffective at delaying or reversing neuropathic complications, the findings from this study also strongly support moderately intense aerobic and/or resistance exercise as relatively safe non-invasive interventions that do not exacerbate peripheral sensory or motor nerve injury in well managed glycemic controlled chronic diabetic neuropathic patients.

As with any patient sample, there are always a subset of individuals that respond to an intervention and those that do not, often referred to as “non-responders.” Our experience with this sample was no different, as we encountered, throughout the course of this study, patients that showed electrodiagnostic improvement in sensory nerve function in response to exercise intervention. In the absence of an objective measure by which “responders” and “non-responders” can be distinguished, we utilized non-parametric analytical approaches to statistically determine qualitative differences among these various data sets. When analyzed in this manner, a 12-week structured exercise program was found to elicit a modest, statistically significant, beneficial effect on sensory nerve fiber function. During the intervening 12-week post-intervention period, even more individuals that had undergone exercise showed improvement on electrodiagnostic findings. When analyzed in an identical non-parametric manner, exercise elicited neither a beneficial or detrimental effect on motor nerve function.

In addition to these qualitative electrodiagnostic analyses, 12 randomized subjects volunteered to be biopsied for determination of epidermal nerve fiber density. The majority (92%) of these patients had diminished levels (<5.4) of epidermal nerve fiber density. Although this sample size is too small to statistically distinguish between exercise intervention types, it is noteworthy that three of six patients (50%) that had undergone exercise intervention exhibited a marked improvement in epidermal nerve fiber density with a fourth patient exhibiting no change. By comparison, two of the three sedentary patients who underwent repeat testing showed a progressive loss of epidermal nerve fiber density with a third having no detectable levels of epidermal nerve fiber density at baseline. These findings infer a restorative effect of exercise on this population of small sensory nerve fibers. The selective effect of exercise on sensory nerve fiber function is not without precedence and may be related to localized increased production by sensory ganglia of numerous neurotrophic (BDNF, NGF, NT-3) and related factors (Cooper et al., 2016).

The aerobic exercise intervention program used in this study is considered moderately challenging, previously shown to increase aerobic capacity to ≥6 METs, a level sufficient to afford cardiorespiratory reserve (fitness) for performing normal activities of daily living (Langbein et al., 2002). This program was well-tolerated by our patients with no exercise-related adverse events encountered. Despite its intensity, there were no immediate or sustained effects of aerobic-, isokinetic strength-, or the combination of aerobic–isokinetic strength training, compared to sedentary controls, on peak patient achieved oxygen uptake. This sample of well-managed glycemic controlled chronic diabetic patients appeared rather resistant to metabolic change and neither benefited or were detrimentally harmed by performing regular-interval exercise intervention. The lack of exercise-improved cardio fitness encountered in this patient population may have limited the impact on our primary electrodiagnostic and secondary quality of life outcome measures in this study. Subjecting diabetic individuals to a more rigorously intense exercise training program is not recommended due to elevated risk of exacerbating associated retinopathic, nephropathic, and neuropathic diabetic complications. Alternatively, extending the length of time patients are subjected to exercise lifestyle intervention beyond 12 weeks of training may prove beneficial to cardio fitness as well as allowing endoneurial vascular remodeling to alleviate ischemic damage to affected peripheral nerves.

As with all randomized clinical trials, this single-site study is not without limitations. Our small sample size coupled with variable electrodiagnostic findings with this chronic patient sample unavoidably limited the statistical power of this study. Since it is well known that exercise improves glycemic management, this study was specifically designed to address and identify whether a specific type of exercise would improve peripheral nerve function among patients exhibiting tight glycemic control. Enrolled patients were therefore clinically well-managed, which may have buffered or masked any exercise-dependent direct effects on peripheral nerve function. The length of time patients were subject to exercise intervention (12 weeks, Fisher et al., 2007) may have also limited our ability to detect objective changes in peripheral nerve function. Longer durations of intermittent exercise may prove more effective at improving peak metabolic parameters among chronic diabetic patients while eliciting endoneurial changes that enhance regenerative repair of distal peripheral nerve fibers. Although our patient sample was comprised of sedentary subjects, we advocated throughout this study lifestyle modifications that included increased physical activity in compliance with recommended standard of care. An objective measure of each patients daily physical activity level, similar to that of HbA1c, would have proved a useful means of normalization.

A structured aerobic exercise program is recommended as a safe well-tolerated adjunctive therapy for the management of diabetic patients with long-standing distal symmetric polyneuropathy. In most patients, a 12-week course of physical exercise, regardless of type, does not appear to adversely alter sensory or motor nerve electrodiagnostic findings. In a subset of patients, a short-term structured program of exercise may selectively improve sensory nerve fiber function. Large-scale exercise lifestyle intervention trials are warranted to further evaluate the impact of aerobic exercise on nerve function in diabetic neuropathic patients.

## Ethics Statement

This study was carried out in accordance with the recommendations of the Edward Hines Jr. VA Hospital human studies subcommittee with written informed consent from all subjects. All subjects gave written informed consent in accordance with the Declaration of Helsinki. The protocol was approved by the Edward Hines Jr. VA Hospital human studies subcommittee.

## Author Contributions

ES, MF, and EC conceived, designed, and implemented the study, participated in data analyses and data interpretation, and contributed to the writing of the original manuscript. CMM was the nurse clinical coordinator for this study. CJ, JB, and CM was the exercise electrophysiologist technicians responsible for training subjects randomized to respective training groups. MF was the neurologist responsible for conducting and collecting all primary outcome neurological EMG data. ES was the principal investigator responsible for overseeing and conducting all administrative aspects of this study and conducting QST measurements.

## Conflict of Interest Statement

The authors declare that the research was conducted in the absence of any commercial or financial relationships that could be construed as a potential conflict of interest.

## Supplementary Material

FIGURE S1 Exercise does not alter SF-36V patient self-reported subscale scores. Data shown are the means ± SD of self-reported responses from patients randomized to sedentary control **(open bars)** or combined exercise **(solid bars)** experimental groups. Indicated subscale scores are expressed as a percentage of weighted score (Supplementary Table S1) determined at entry into the study (baseline), immediately following intervention, and again at 12-week post-intervention, as indicated. The total number of patients that completed the SF-36V questionnaire at baseline (sedentary controls, *N* = *12*; exercise, *N* = *33*), immediately following intervention (sedentary controls, *N* = *10*; exercise, *N* = *30*), and at 12-week post-intervention (sedentary controls, *N* = *9*; exercise, *N* = *28*) were group analyzed. At all three time points evaluated, there was no significant differences observed within and across experimental groups. Non-parametric data were analyzed by ANOVA on ranks (Kruskal–Wallis test). Note that one patient randomized to exercise did not complete the 12-week post SF-36V questionnaire.

TABLE S1 SF-36V Health survey health domain scales.

TABLE S2 RAND 36-item health survey scoring template.

TABLE S3 Relative effect of exercise type on self-reported health status using the SF-36V questionnaire.
