# Supplementary material for: Randomized Controlled Trial of Physical Exercise in Diabetic Veterans With Length-Dependent Distal Symmetric Polyneuropathy
Source: Front Neurosci. 2019 Feb 11;13:51. doi: 10.3389/fnins.2019.00051 (PMC6379046; doi:10.3389/fnins.2019.00051)

Physical Functioning

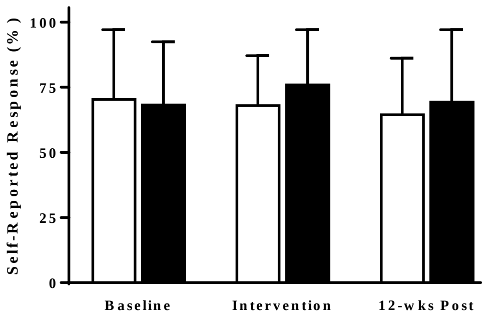

Physical Limitation

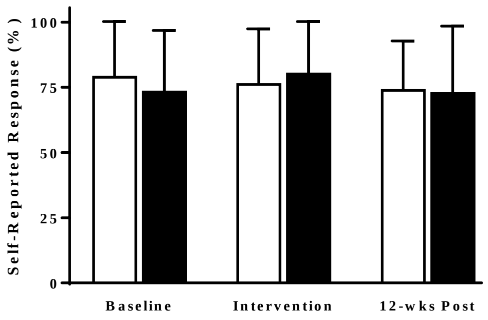

Bodily Pain

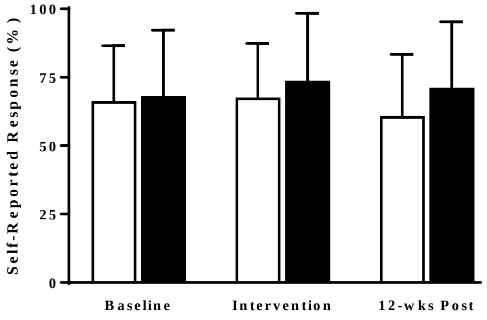

General Health Perceptions

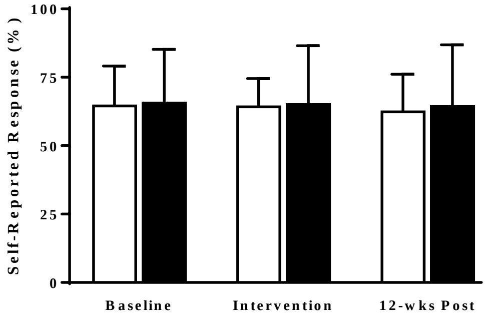

Energy/Vitality

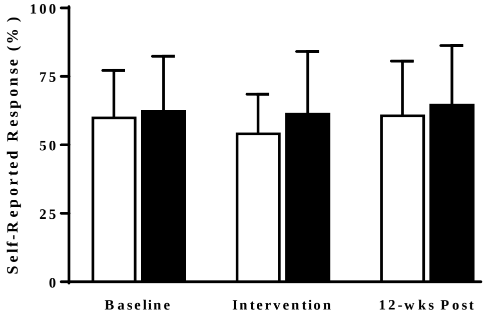

Mental Health

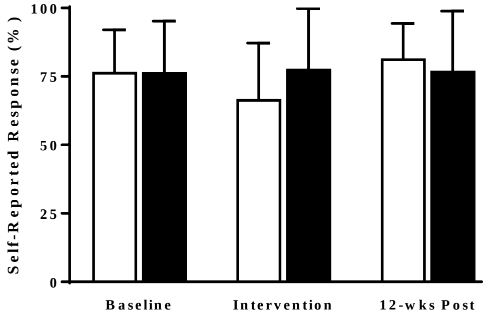

Social Functioning

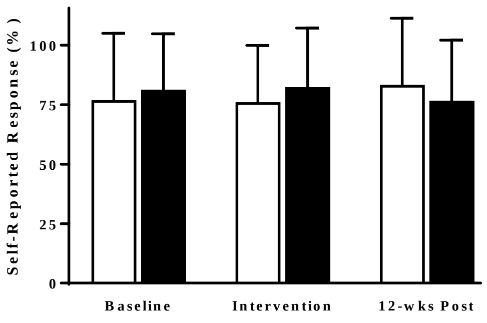

Emotional Limitations

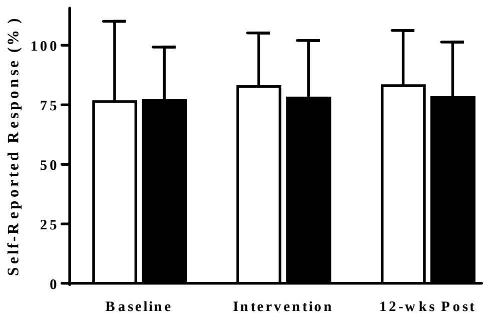

Supplement: FIGURE S1 — Exercise does not alter SF-36V patient self-reported subscale scores. Data shown are the means ± SD of self-reported responses from patients randomized to sedentary control (open bars) or combined exercise (solid bars) experimental groups. Indicated subscale scores are expressed as a percentage of weighted score (Supplementary Table S1) determined at entry into the study (baseline), immediately following intervention, and again at 12-week post-intervention, as indicated. The total number of patients that completed the SF-36V questionnaire at baseline (sedentary controls, N = 12; exercise, N = 33), immediately following intervention (sedentary controls, N = 10; exercise, N = 30), and at 12-week post-intervention (sedentary controls, N = 9; exercise, N = 28) were group analyzed. At all three time points evaluated, there was no significant differences observed within and across experimental groups. Non-parametric data were analyzed by ANOVA on ranks (Kruskal–Wallis test). Note that one patient randomized to exercise did not complete the 12-week post SF-36V questionnaire. [file Data_Sheet_1.PDF]
