# Supplementary material for: Randomized Controlled Trial of Physical Exercise in Diabetic Veterans With Length-Dependent Distal Symmetric Polyneuropathy
Source: Front Neurosci. 2019 Feb 11;13:51. doi: 10.3389/fnins.2019.00051 (PMC6379046; doi:10.3389/fnins.2019.00051)
Supplement: TABLE S1 — SF-36V Health survey health domain scales. [file Data_Sheet_2.PDF]

Table ***S1.*** *SF-36V Health Survey Health Domain Scales.*

Scale	*Items*	*Number of Items*	*Total Score Possible*	*Abbreviated Item Content*
Physical Functioning	2a-2j	10	1000	Daily Activities
Role Limitations Due to Physical Problems	3a-d	4	400	Physical Limitations to Daily Activities over the past 4-weeks
Bodily Pain	6 & 7	2	200	Bodily Pain Experienced in the past 4-weeks and how this pain limits your daily activities
General Health Perceptions	1 & 10a-10d	5	500	General Well Being: Health
Energy/Vitality	8a, 8e, 8g, 8i	4	400	General Well Being: Energy
Social Functioning	5, 9	2	200	Physical or Emotional Health-Associated Limitations of Social Activities
Role Limitations Due to Emotional Problems	4a-c	3	300	Emotional Limitations to Daily Activities over the past 4-weeks
Mental Health	8b-d, 8f, 8h	5	500	General Well Being: Emotional
**Summary Measures**				
Physical Component Score (PCS)	11	1	100	Physical Health Compared to One-Year Ago
Mental Component Score (MCS)	12	1	100	Emotional Health Compared to One-Year Ago

All questions scored on a scale of 0-100, with 100 representing the highest level of functioning possible. Aggregate scores are compiled as a percentage of the total points possible using the RAND 36-item health survey scoring table (see supplemental *Table S2*).
