# Supplementary material for: Randomized Controlled Trial of Physical Exercise in Diabetic Veterans With Length-Dependent Distal Symmetric Polyneuropathy
Source: Front Neurosci. 2019 Feb 11;13:51. doi: 10.3389/fnins.2019.00051 (PMC6379046; doi:10.3389/fnins.2019.00051)
Supplement: TABLE S2 — RAND 36-item health survey scoring template. [file Data_Sheet_3.PDF]

Table ***S2.*** *RAND 36-item health survey scoring template.*

SF-36V Question	***Original Response Options***	***Weighted Score***
1, 3, 4, 5, 7, 10b, 10d, 11, 12	1<br>2<br>3<br>4<br>5	100<br>75<br>50<br>25<br>0
2	1<br>2<br>3	0<br>50<br>100
6, 8a, 8d, 8e, 8h,	1<br>2<br>3<br>4<br>5<br>6	100<br>80<br>60<br>40<br>20<br>0
8b, 8c, 8f, 8g, 8i,	1<br>2<br>3<br>4<br>5<br>6	0<br>20<br>40<br>60<br>80<br>100
9, 10a, 10c,	1<br>2<br>3<br>4<br>5	0<br>25<br>50<br>75<br>100
