# Supplementary material for: Randomized Controlled Trial of Physical Exercise in Diabetic Veterans With Length-Dependent Distal Symmetric Polyneuropathy
Source: Front Neurosci. 2019 Feb 11;13:51. doi: 10.3389/fnins.2019.00051 (PMC6379046; doi:10.3389/fnins.2019.00051)
Supplement: TABLE S3 — Relative effect of exercise type on self-reported health status using the SF-36V questionnaire. [file Data_Sheet_4.PDF]

Table S3. *Relative effect of exercise type on self-reported health status using the SF-36V questionnaire.*

SubScale	Sedentary	Aerobic	Strength	Aerobic + Strength	*p*
**Physical Functioning**					
**Baseline**	70.8 ± 26.3 (12)	73.3 ± 13.7 (11)	64.5 ± 27.4 (11)	68.2 ± 28.7 (11)	*0.88*
**Intervention**	68.5 ± 18.6 (10)	79.8 ± 16.5 (11)	67.0 ± 23.4 (10)	82.8 ± 20.6 (9)	*0.22*
p	*1.00*	*0.37*	*1.00*	*0.62*	
**12-wks Post**	65.0 ± 21.1 (9)	64.1 ± 30.6 (11)	64.0 ± 28.1 (10)	87.1 ± 11.9 (7)	*0.11*
p	*1.00*	*0.61*	*1.00*	*1.00*	
**Physical Limitations**					
**Baseline**	79.4 ± 20.8 (12)	71.1 ± 22.9 (11)	72.0 ± 27.8 (11)	78.1 ± 19.6 (11)	*0.85*
**Intervention**	76.6 ± 20.8 (10)	80.9 ± 20.9 (11)	76.6 ± 23.1 (10)	84.3 ± 15.0 (9)	*0.89*
p	*1.00*	*0.54*	*1.00*	*1.00*	
**12-wks Post**	74.3 ± 18.5 (9)	80.9 ± 17.2 (11)	61.7 ± 28.3 (10)	77.1 ± 29.2 (7)	*0.41*
p	*1.00*	*1.00*	*0.71*	*1.00*	
**Bodily Pain**					
**Baseline**	66.3 ± 20.2 (12)	69.1 ± 22.6 (11)	62.7 ± 21.8 (11)	72.1 ± 28.9 (11)	*0.70*
**Intervention**	67.6 ± 19.8 (10)	73.8 ± 25.6 (11)	76.2 ± 25.3 (10)	70.3 ± 25.9 (9)	*0.61*
p	*1.00*	*1.00*	*0.48*	*1.00*	
**12-wks Post**	60.9 ± 22.5 (9)	78.2 ± 26.5 (11)	67.3 ± 19.9 (10)	65.4 ± 26.8 (7)	*0.25*
p	*1.00*	*1.00*	*1.00*	*1.00*	
**General Health Perceptions**					
**Baseline**	65.0 ± 14.1 (12)	63.5 ± 17.8 (11)	63.6 ± 18.0 (11)	70.9 ± 22.3 (11)	*0.70*
**Intervention**	64.7 ± 9.8 (10)	62.5 ± 21.3 (11)	63.2 ± 27.6 (10)	71.3 ± 10.6 (9)	*0.66*
p	*1.00*	*1.00*	*1.00*	*1.00*	
**12-wks Post**	62.9 ± 13.2 (9)	67.9 ± 22.0 (11)	59.0 ± 27.4 (10)	67.9 ± 14.4 (7)	*0.79*
p	*1.00*	*1.00*	*1.00*	*1.00*	

**Energy/Vitality**					
**Baseline**	60.4 ± 16.8 (12)	60.6 ± 23.6 (11)	60.0 ± 16.0 (11)	67.3 ± 20.3 (11)	*0.83*
**Intervention**	54.5 ± 14.0 (10)	58.7 ± 24.0 (11)	62.0 ± 26.9 (10)	65.0 ± 16.4 (9)	*0.51*
p	*1.00*	*1.00*	*1.00*	*1.00*	
**12-wks Post**	61.1 ± 19.5 (9)	64.1 ± 20.6 (11)	61.5 ± 25.1 (10)	71.4 ± 30.2 (7)	*0.73*
p	*1.00*	*1.00*	*1.00*	*1.00*	
**Social Functioning**					
**Baseline**	76.9 ± 28.1 (12)	74.9 ± 26.9 (11)	87.4 ± 19.5 (11)	81.6 ± 24.0 (11)	*0.60*
**Intervention**	76.0 ± 23.9 (10)	81.7 ± 23.3 (11)	76.1 ± 32.1 (10)	90.1 ± 16.5 (9)	*0.54*
p	*1.00*	*1.00*	*1.00*	*0.92*	
**12-wks Post**	83.3 ± 28.0 (9)	72.5 ± 23.7 (11)	73.7 ± 31.5 (10)	87.3 ± 17.9 (7)	*0.57*
p	*0.87*	*1.00*	*1.00*	*1.00*	
**Emotional Limitations**					
**Baseline**	76.9 ± 33.2 (12)	73.2 ± 20.5 (11)	81.7 ± 22.0 (11)	77.0 ± 24.4 (11)	*0.80*
**Intervention**	83.2 ± 22.0 (10)	80.1 ± 20.7 (11)	70.7 ± 31.0 (10)	85.0 ± 16.7 (9)	*0.75*
p	*1.00*	*1.00*	*1.00*	*1.00*	
**12-wks Post**	83.6 ± 22.6 (9)	85.2 ± 15.1 (11)	74.0 ± 28.3 (10)	74.3 ± 25.1 (7)	*0.85*
p	*1.00*	*1.00*	*1.00*	*1.00*	
**Mental Health**					
**Baseline**	76.7 ± 15.3 (12)	79.3 ± 13.8 (11)	78.2 ± 21.8 (11)	72.0 ± 20.6 (11)	*0.75*
**Intervention**	66.8 ± 20.4 (10)	76.4 ± 18.5 (11)	72.8 ± 30.5 (10)	85.1 ± 13.7 (9)	*0.24*
p	*0.63*	*1.00*	*1.00*	*0.46*	
**12-wks Post**	81.6 ± 12.8 (9)	81.5 ± 14.9 (11)	69.4 ± 29.9 (10)	81.1 ± 16.6 (7)	*0.93*
p	*0.20*	*1.00*	*1.00*	*1.00*	

Data shown are the means ± SD of (N) patients. These non-parametric data were analyzed using a Kruskal-Wallis test with Dunn's multiple comparison analysis. For within group analyses, baseline values are compared with intervention; intervention values are compared with 12-week post intervention values.
